# Prediction of risk factors for intraoperative hypotension during general anesthesia undergoing carotid endarterectomy

**DOI:** 10.3389/fneur.2022.890107

**Published:** 2022-09-06

**Authors:** Yitong Jia, Guang Feng, Zheng Wang, Yao Feng, Liqun Jiao, Tian-Long Wang

**Affiliations:** ^1^Department of Anesthesiology, Xuanwu Hospital, Capital Medical University, Beijing, China; ^2^Department of General Surgery, Xuanwu Hospital, Capital Medical University, Beijing, China; ^3^Department of Neurosurgery, Xuanwu Hospital, Capital Medical University, Beijing, China

**Keywords:** carotid endarterectomy, intraoperative hypotension, prediction of risk factors, general anesthesia, retrospective study

## Abstract

**Objective:**

Carotid endarterectomy (CEA) has been considered as “gold standard” treatment for patients with significant carotid stenosis Intra-operative hypotension was a risk factor for post-operative complications in patients undergoing CEA. This study aimed to investigate the predictors for intra-operative hypotension during CEA.

**Methods:**

This retrospective study included consecutive patients underwent CEA from June 1, 2020 to May 31, 2021 in the neurosurgery department of Xuanwu Hospital, Capital Medical University. The intraoperative hypotension was defined as blood pressure (BP) of 20% below standard value for longer than 5 min. Univariable and multivariable analyses were performed to identify the prediction of risk factors for intraoperative hypotension.

**Results:**

Overall, 367 patients were included, and 143 (39.0%) patients had hypotension during CEA procedure. Univariate analysis indicated Grade 3 hypertension (*P* = 0.002), peripheral artery disease (*P* = 0.006) and shunting (*P* = 0.049) were associated with occurrence of intraoperative hypotension during CEA procedure. On multivariable analysis, Grade 3 hypertension (*P* = 0.005), peripheral artery disease (*P* = 0.009), and shunting (*P* = 0.034) were all found to be independent predicting factors of hypotension during the CEA process.

**Conclusion:**

Intraoperative hypotension is a dynamic phenomenon may be affected by patients with grade 3 hypertension, peripheral artery disease and intra-operative shunting. It is necessary to pay special attention to these patients, both intraoperatively and postoperatively, to improve the final clinical outcome.

## Introduction

Stroke is a leading cause of death worldwide, claiming the lives of approximately 12 million people each year (1). Atherosclerosis of the carotid artery is a common cause of stroke and gives rise to enormous morbidity with long-term disability and financial burden besides death (2). Drug therapy, carotid endarterectomy (CEA), carotid artery stenting (CAS) and transcarotid artery revascularization (TCAR) are the four treatment options currently available. Although the other three methods have been improved and developed in recent years, CEA has been considered as “gold standard” treatment for patients with significant carotid stenosis to prevent future cerebrovascular events for many years (3, 4).

Hemodynamic disturbance, such as bradycardia, extreme hypotension and hyperperfusion, is an important mechanism for procedural stroke in both CAS and CEA. Cerebral hyperperfusion occasionally causes unilateral headache, seizures, and focal symptoms that are very occasionally accompanied by intracerebral hemorrhage (5), attracting a large number of researchers to conduct studies (6–8). Strict perioperative arterial blood pressure (BP) control has been advocated to preserve adequate cerebral perfusion during CEA (9). However, both the baroreflex and cerebral autoregulation can be impaired in carotid atherosclerosis (10, 11), which may lead to cerebral hypoperfusion even without systemic hemodynamic disturbances. Cerebral hypoperfusion is related to subsequent ischemia in the brain, or the inability to flush the artery-to-artery emboli well during CEA (12, 13). Kobayashi et al. (14) prevented the postoperative development of new cerebral ischemic lesions on diffusion weighted imaging (DWI) by intentional hypertension during dissection of the carotid artery in CEA.

Recently, Rots et al. (15) found that intra-operative hypotension was a risk factor for post-operative silent brain ischemia in patients with pre-operative hypertension undergoing CEA. Previous studies confirmed that post-operative silent brain ischemia detected by DWI was associated with cognitive impairment and higher risk of future cerebrovascular events (16–18). To our knowledge, few studies have explored the predictors for intra-operative hypotension during CEA. Therefore, the aims of this research were to investigate these predictors based on clinical features, lesion characteristics and intraoperative related information, to alert the surgeon to notice the phenomenon of intraoperative hypotension.

## Methods

### Study design and patient population

A retrospective analysis was performed with the data extracted from the medical records who underwent CEA in general anesthesia in the neurosurgery department of Xuanwu Hospital, Capital Medical University from June 1, 2020 to May 31, 2021. Our hospital's institutional review board authorized this study protocol, and patients' consent was not required because of the retrospective nature of this study.

Patients with a recent symptomatic carotid stenosis of 50~99% or asymptomatic carotid stenosis of 70~99% according to the method of carotid stenosis measurement by the North American Symptomatic Carotid Endarterectomy Trial (NASCET) with CEA therapy were included in this study. Furthermore, patients with carotid web treated with CEA were also included in this study. Duplex ultrasound, CT angioplasty (CTA) or digital subtraction angiography (DSA) were used to determine the degree of stenosis. The variables included patients' demographic characteristics (e.g., age, sex, comorbidities, degree of stenosis, plaque characteristics, operation side and intraoperative parameters).

### CEA procedure

All of the operations were performed under general anesthesia by neurosurgeons with transcranial Doppler (TCD) used throughout to monitor the procedure. The surgical techniques, standard CEA, eversion CEA or patch CEA, shunt or not, were determined by surgeons (19).

### Outcome definition

The primary outcome variable for our analysis was the intraoperative hypotension. The BP of 20% below the standard value was defined as intraoperative hypotension. Simultaneously, the duration of hypotension must be longer than 5 min (15, 20). The standard BP was based on the patient's usual BP and the middle cerebral artery blood flow value measured by transcranial Doppler (TCD). BP was obtained through invasive arterial pressure during procedure. For the clinical outcomes, the definition of myocardial infarction, stroke and death was carried over from our previously published article (21). Cerebral hyperperfusion syndrome is defined as a symptom including a severe ipsilateral headache, focal neurological deficits, intracerebral hemorrhage, and occasionally includes seizures or encephalopathy (22). The composite outcome covers all the adverse outcomes mentioned above.

### Statistics

Continuous variables were presented as mean ± standard deviation or median (interquartile range, IQR) according to whether it conformed to the normal distribution. Categorical variables were reported as number (and proportion) of patients. Differences in continuous parameters were calculated with a student *t*-test or a Mann-Whitney *U*-test, as appropriate. The Chi square test or Fisher's exact test as appropriate would be used to compare categorical variables. A *P* < 0.05 was considered statistically significant. Univariate analysis with statistically significant value or some important parameter would be entered into the multivariate logistic regression analysis to determine the independent predictors. SPSS statistical software version 23.0 (SPSS, New York, USA) was used for the data analysis.

## Results

### Patient characteristics and clinical outcomes

**Figure 1:**
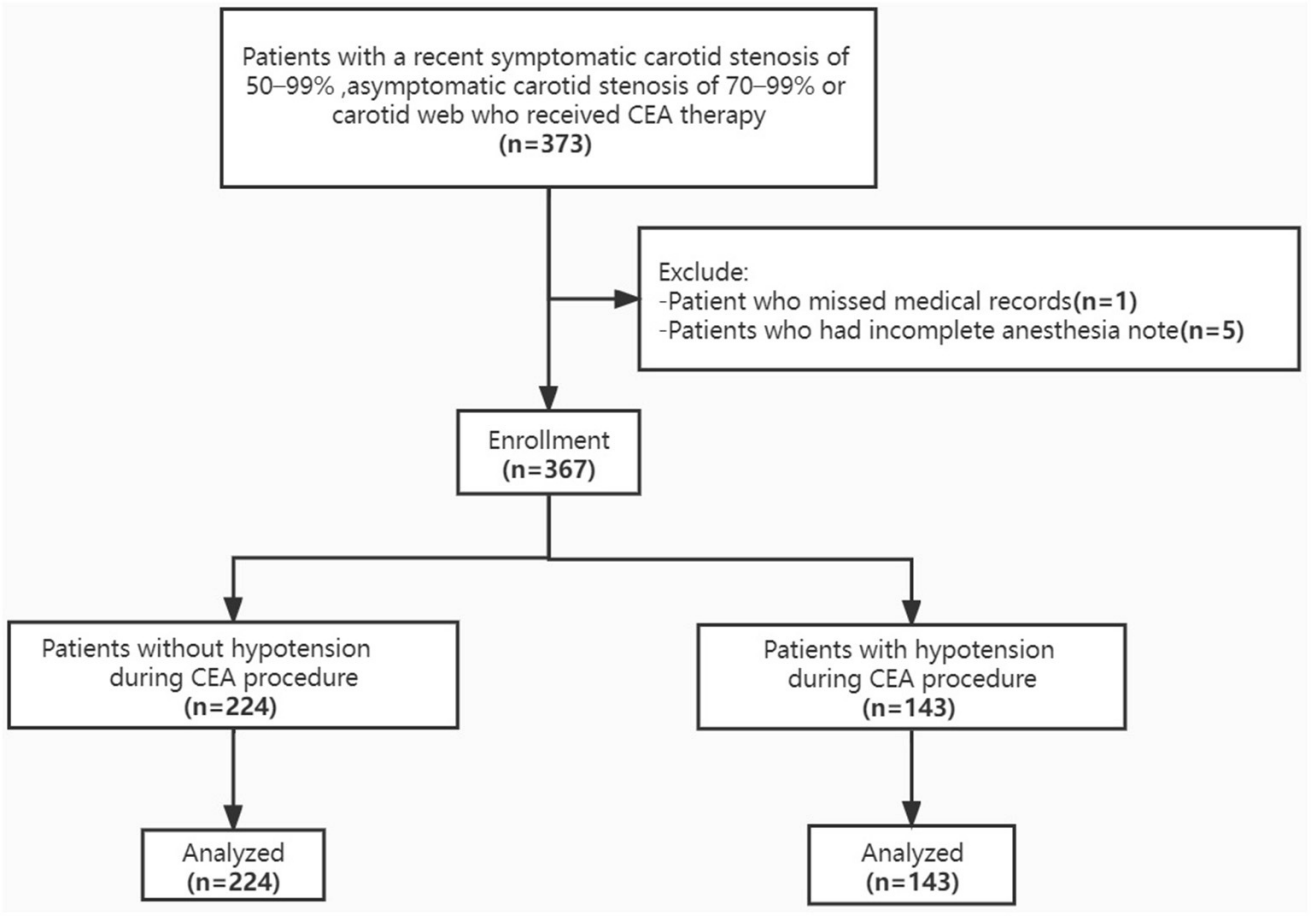
Flow diagram of the study populations.

A total of 367 patients underwent CEA procedures with comprehensive medical record were included in this study (Figure 1). Median age of patients was 64 years (IQR 60, 68), which of 86.9% were man. The types of lesions were divided into carotid webs, in-stent restenosis and atherosclerotic plaques, and the proportions were 1.9, 3.5, and 94.6%, respectively. Moderate stenosis, severe stenosis, near-occlusion and total occlusion of carotid artery accounted for 9.8, 76.0, 2.7, and 11.4%, respectively. Detailed patient characteristics were shown in Table 1.

**Table 1:** Baseline characteristics of patients' cohort.

**Characteristics**	**Total,** ***n* = 367, (%)**	**Hypotension group,** ***n* = 143, (%)**	**Non-hypotension group,** ***n* = 224, (%)**	***p*-value**
Age, years (median, IQR)	64 (60, 68)	65 (60, 69)	64 (59, 68)	0.203
Sex, male	319 (86.9)	126 (88.1)	193 (86.2)	0.589
Hypertension	261 (71.1)	98 (68.5)	163 (72.8)	0.383
Grade 3 hypertension^†^	14 (3.8)	11 (7.7)	3 (1.3)	0.002
Diabetes mellitus	116 (31.6)	47 (32.9)	69 (30.8)	0.678
Arrhythmia	15 (4.1)	4 (2.8)	11 (4.9)	0.319
Coronary artery disease	56 (15.3)	19 (13.3)	37 (16.5)	0.401
Hyperlipemia	208 (56.7)	81 (57.3)	127 (56.3)	0.837
Cerebral infarction	213 (58.0)	88 (61.5)	125 (55.8)	0.278
Peripheral artery disease	17 (4.6)	12 (8.4)	5 (2.2)	0.006
Chronic kidney disease	10 (2.7)	3 (2.1)	7 (3.1)	0.746
Smoking	215 (58.6)	86 (60.1)	129 (57.6)	0.629
Drinking	171 (46.6)	60 (66.6)	111 (49.6)	0.155
Lesion characteristics				
Lesion type				
Carotid web	7 (1.9)	1 (0.7)	6 (2.7)	0.276
In-stent stenosis	13 (3.5)	7 (5.1)	6 (2.7)	
Plaque	347 (94.6)	135 (94.4)	212 (94.6)	
Lesion side, left	189 (51.5)	74 (51.7)	115 (51.3)	0.939
Degree of stenosis				
Moderate	36 (9.8)	13 (9.1)	23 (10.3)	0.170
Severe	279 (76.0)	103 (72.0)	176 (78.6)	
Near-total occlusion	10 (2.7)	4 (2.8)	6 (2.7)	
Total occlusion	42 (11.4)	23 (16.1)	19 (8.5)	
Contralateral stenosis (≥50%)	71 (19.3)	32 (22.4)	39 (17.4)	0.240
Operation information				
Procedure duration (IQR)	133 (103, 188)	132 (102, 190)	133 (104, 186)	0.912
Anesthesia method				
Intravenous anesthesia	301 (82.0)	122 (85.3)	179 (79.9)	0.189
Intravenous combined with respiration anesthesia	66 (18.0)	21 (20.1)	45 (14.7)	
Clamping time, min (*n* = 347, median, IQR)	34 (24,48)	34 (25, 51)	34 (24, 46)	0.668
Shunting	46 (12.5)	24 (16.8)	22 (9.8)	0.049

† This blood pressure was measured in ward and either the systolic blood pressure ≥180 mmHg, or the diastolic blood pressure ≥110 mmHg.

One hundred and forty-three (39.0%) patients had hypotension during CEA procedure. In Table 2, we performed analyses specifically for clinical outcomes. Adverse outcomes occurred in 16 cases, included 3 myocardial infarctions, 6 strokes, 1 death, and 6 hyperperfusion syndromes. There were significant differences in the occurrence of adverse composite outcome (*P* < 0.001), stroke (*P* = 0.003) and hyperfusion syndrome (*P* = 0.036) in patients with or without intraoperative hypotension.

**Table 2:** Clinical outcome stratified for patients with hypotension or not.

**Outcomes**	**Total, *n* = 367, (%)**	**Non-hypotension group, *n* = 224, (%)**	**Hypotension group, *n* = 143, (%)**	***p*-value**
Composite outcome	16 (4.4)	1 (0.4)	15 (10.4)	<0.001
Myocardial infarction	3 (0.8)	0 (0)	3 (2.1)	0.060
Stroke	6 (1.6)	0 (0)	6 (4.9)	0.003
Death	1 (0.3)	0 (0)	1 (0.7)	0.392
Hyperperfusion syndrome	6 (1.6)	1 (0.4)	5 (3.5)	0.036

### Univariate analysis

The results of the univariate analysis of patient demographics, comorbidities, lesion characteristics and operation information were shown in Table 1. Grade 3 Hypertension (7.7 vs. 1.3%, *P* = 0.002), peripheral artery disease (8.4 vs. 2.2%, *P* = 0.006) and intra-operative shunting (16.8 vs. 9.8%, *P* = 0.049) were associated with occurrence of intraoperative hypotension during CEA procedure.

### Multivariate analysis

Multivariate logistic regression analysis was carried out to identify independent prediction of high risk factors for intraoperative hypotension during CEA procedure (Table 3). According to the results of univariate analysis. We included Grade 3 hypertension, peripheral artery disease, shunting and age into our multivariate analysis. Grade 3 hypertension (*P* = 0.005), peripheral artery disease (*P* = 0.009), and shunting *P* = 0.034) were all found to be independent predicting factors of hypotension during the CEA process.

**Table 3:** Multivariate logistic regression of independent risk factors of hypotension during CEA procedure.

**Characteristics**	**OR**	**95% CI**	***p*-value**
Age	1.029	0.999–1.059	0.062
Peripheral artery disease	4.286	1.431–12.838	0.009
Grade 3 hypertension	6.690	1.773–25.246	0.005
Shunting	1.978	1.051–3.721	0.034

## Discussion

This study explored the predictors for intraoperative hypotension during CEA procedure. The preliminary results suggested that grade 3 hypertension, peripheral artery disease and shunting were relevant with intraoperative hypotension occurrence.

There is a lack of reports specifically assessing the predictors of intraoperative hypotension in patients undergoing CEA. Because of different definitions mentioned in the literature, it is very difficult to determine the exact incidence of intraoperative hypotension during CEA procedure. The incidence of intraoperative hypotension reported by Sposato et al. (20) was 8.6%, while reported by Rots et al. (15) was as high as 96.4%. In this study, the incidence of intraoperative is approximately 39.0%. Intraoperative hypotension is most likely caused by the impaired brain self-regulatory function of the patient. Intraoperative hypotension was reported with the occurrence of new onset atrial fibrillation, adverse outcomes (perioperative ischemic and myocardial infarction) and postoperative silent brain infarction (15, 20). Our study also found that intraoperative hypotension could lead to adverse events, such as myocardial infarction, stroke, and hyperperfusion syndrome. Besides, intraoperative hypotension was related to postoperative hematoma formation due to a false impression that adequate hemostasis had been achieved (23). Therefore, intraoperative hypotension during CEA procedure should not be ignored. Although there are many reports focusing on postoperative hypertension which could lead to cerebral hyperperfusion syndrome (9).

Cardiovascular lability is of great problem in hypertensive patients undergoing anesthesia and surgery. These patients are prone to episodes of hypotension and hypertension during the operation (24, 25). CEA surgery is more complicated for hemodynamic changes, which are reflected in carotid cross-clamping, general anesthesia, and surgical manipulation of the carotid bifurcation (26). In this study, we found that patients with Grade 3 hypertension and peripheral artery disease had a higher incidence rate of intraoperative hypotension. Patients with high baseline BP may be at risk of brain ischemia because of the failure of auto-regulatory mechanisms. Negative feedback system can assist the body in stabilizing BP and cerebral blood flow under physiological conditions (27). Previous studies confirmed that patients with carotid artery disease may have impaired cerebral autoregulation (28) and additionally have attenuated baroreflex sensitivity because of reduced distensibility of the carotid bulbs influenced by atherosclerosis (10). The presence of peripheral artery disease is also an important marker for systemic atherosclerosis involving the coronary, cerebral, and renal vascular territories (29). Severe atherosclerosis could make the arterial wall less deformable, thereby impairing patients' BP-regulating capacity (30). Once the baroreflex-mediated BP regulation suffered, BP variability would increase, causing cardiovascular events (31). Patients with grade 3 hypertension are more likely to have impaired the body's regulatory functions. Therefore, BP may be more unstable when undergoing surgery, necessitating more attention from anesthesiologists and surgeons.

Multivariate analysis also showed shunting was an independent predictor for intraoperative hypotension. Although shunting is a useful technique to maintain blood flow in those patients with contralateral carotid stenosis or a compromised Circle of Willis, it is not an entirely benign intervention. Complications such as air or plaque embolization, intimal tears, and carotid dissection may be due to shunt insertion (26). We guess that on the one hand, the bypass operation can physically stimulate the carotid sinus, and on the other hand, it increases the difficulty of the operation for the operator, thereby affecting patients' BP. What's more, shunting is selective for patients with poor mean flow velocity of the ipsilateral middle cerebral artery when clamping carotid artery in our center. There may also be selection bias in these patients with undercompensated collaterals. Previous large sample study (32) showed no significant difference of stroke or death for routine shunting compared with selective shunting or never shunting. Therefore, skill in shunting may be an important influencing factor. When inserting a shunt, we need to operate gently and pay attention to the patient's BP.

In some centers, regional anesthesia is used during CEA. In 2008, a randomized controlled trial of general anesthesia vs. local anesthesia for carotid surgery (GALA) (33) aimed to explore the composite outcome within 30 days about these two types of anesthesia. However, the GALA trial didn't show a definite difference in outcomes between general and local anesthesia for CEA. Jacques et al. (34) found that less intraoperative hypotension and vasopressor requirement for regional anesthesia compared with general anesthesia when performing CEA. More recently, Leblanc et al. (35) also found low incidence rate of severe hypotension with regional anesthesia. Larger sample sizes are still needed to further clarify this association.

There are certain limitations. First, this was a retrospective review of single-center clinical data, so some uncertainty may exist. Second, the hazards related to intraoperative hypotension were based on previous literatures, and the predictors were carried out on this basis. Third, the definition of intraoperative hypotension varied across the literature. And in this study, we put forward our own definition of the concept of intraoperative hypotension. Despite its limitations, the predictors identified by this study may prompt a re-evaluation of surgical practice patterns, and target areas for further research. In particular, prospective studies are needed to confirm these findings.

## Conclusions

In light of the foregoing, we conclude that intraoperative hypotension is a dynamic phenomenon affected by many factors. The presence of grade 3 hypertension, peripheral artery disease and shunting were all found to be independent predictors for intraoperative hypotension in our study. To prevent postoperative complications, it is important to determine predictors for intraoperative hypotension in order to regulate it properly. It is necessary to pay special attention to these patients, both intraoperatively and postoperatively, to improve the final clinical outcome.

## Data availability statement

The raw data supporting the conclusions of this article will be made available by the authors, without undue reservation.

## Ethics statement

Ethical review and approval was not required for the study on human participants in accordance with the local legislation and institutional requirements. Written informed consent for participation was not required for this study in accordance with the national legislation and the institutional requirements.

## Author contributions

YJ, GF, and T-LW made substantial contributions to conception and design. YJ and ZW have been involved in acquisition, analysis, and interpretation of data. YJ and T-LW made substantial contributions to manuscript preparation, editing, and review. GF and LJ made contributions to English language editing. YF and LJ have given final approval of the version to be published and agreed to be accountable for all aspects of the work in ensuring that questions related to the accuracy or integrity of any part of the work are appropriately investigated and resolved.

## Funding

This study was funded and sponsored by Beijing Municipal Medical Science Institute-Public Welfare Development Reform Pilot Project (Capital Medical Research No. 2019-2).

## Conflict of interest

The authors declare that the research was conducted in the absence of any commercial or financial relationships that could be construed as a potential conflict of interest.

## Publisher's note

All claims expressed in this article are solely those of the authors and do not necessarily represent those of their affiliated organizations, or those of the publisher, the editors and the reviewers. Any product that may be evaluated in this article, or claim that may be made by its manufacturer, is not guaranteed or endorsed by the publisher.
